# A Global Metabolic Shift Is Linked to *Salmonella* Multicellular Development

**DOI:** 10.1371/journal.pone.0011814

**Published:** 2010-07-27

**Authors:** Aaron P. White, Aalim M. Weljie, Dmitry Apel, Ping Zhang, Rustem Shaykhutdinov, Hans J. Vogel, Michael G. Surette

**Affiliations:** 1 Department of Microbiology and Infectious Diseases, University of Calgary, Calgary, Canada; 2 Department of Biological Sciences, University of Calgary, Calgary, Canada; Cinvestav, Mexico

## Abstract

Bacteria can elaborate complex patterns of development that are dictated by temporally ordered patterns of gene expression, typically under the control of a master regulatory pathway. For some processes, such as biofilm development, regulators that initiate the process have been identified but subsequent phenotypic changes such as stress tolerance do not seem to be under the control of these same regulators. A hallmark feature of biofilms is growth within a self-produced extracellular matrix. In this study we used metabolomics to compare *Salmonella* cells in rdar colony biofilms to isogenic *csgD* deletion mutants that do not produce an extracellular matrix. The two populations show distinct metabolite profiles. Even though CsgD controls only extracellular matrix production, metabolite signatures associated with cellular adaptations associated with stress tolerances were present in the wild type but not the mutant cells. To further explore these differences we examine the temporal gene expression of genes implicated in biofilm development and stress adaptations. In wild type cells, genes involved in a metabolic shift to gluconeogenesis and various stress-resistance pathways exhibited an ordered expression profile timed with multicellular development even though they are not CsgD regulated. In *csgD* mutant cells, the ordered expression was lost. We conclude that the induction of these pathways results from production of, and growth within, a self produced matrix rather than elaboration of a defined genetic program. These results predict that common physiological properties of biofilms are induced independently of regulatory pathways that initiate biofilm formation.

## Introduction 

Bacteria frequently grow in multicellular communities that can exhibit complex phenotypes. How the cells organize and how these phenotypes are regulated is of fundamental importance in many areas of microbiology. Biofilms are examples of bacterial multicellular behavior. Biofilm is an umbrella term describing the growth of bacterial cells encased within an extracellular matrix usually in association with surfaces. Biofilms are thought to be the most prevalent form of bacterial life in nature and represent an evolutionarily conserved strategy for survival and persistence [1]. In addition, they are implicated in >60% of human infectious diseases with tremendous health and economic impacts [2]. Characteristics of biofilms include high cell densities, nutrient limitation, and matrix components that serve to link individual cells together [3]. Cells within a biofilm also display remarkable stress tolerance including reduced susceptibility to antibiotics. Many factors contribute to this elevated resistance including regulatory mechanisms [4], [5] and physical and chemical protection by protein, polysaccharide or nucleic acid polymers in the extracellular matrix [1]. These polymers can also aid survival by nutrient trapping, buffering and water retention [6]. The stress tolerance of biofilms is a common feature independent of how they are formed. However, it remains to be answered how biofilms grown under different conditions show common phenotypes without a coordinating regulatory pathway.

For *Salmonella*, the best characterized biofilm state is a coordinated multicellular behaviour termed the rdar morphotype [7], [8]. The formation of rdar (red, dry and rough) colonies is marked by a shift from smooth to an aggregative morphology which results from the ordered production of extracellular matrix components [9]. The matrix in rdar morphotype colonies is primarily comprised of curli fimbriae (also called thin aggregative fimbriae or Tafi) and several exopolysaccharides (EPS), including cellulose and an O-antigen capsule [10], [11], [12]. These polymers are produced in response to starvation [13], triggered through activation of σ^S^ (RpoS), the sigma factor that regulates the general stress response [14], [15], and ultimately CsgD, the main transcriptional regulator of the rdar morphotype [8], [12]. CsgD activation is controlled by a complex regulatory cascade leading to increased intracellular levels of bis (3′-5′) cyclic dimeric guanosine monophosphate (c-di-GMP) [16], [17], [18]. CsgD controls aggregation by directly stimulating transcription of *csgBAC*, encoding the curli structural proteins, and *adrA*, encoding a diguanylate cyclase that activates cellulose production [12], [13]. The rdar morphotype polymers provide a survival advantage through enhanced resistance to desiccation and disinfection [9], [19], [20], allowing cells to survive for up to several years [21]. The rdar morphotype is hypothesized to represent a critical state in the transmission of *Salmonella* between hosts [13], [22].

Microarrays [23], [24], [25], mutagenesis [26], [27] and proteomics [28], [29], along with many other approaches, have been successfully used to identify differences between planktonic and biofilm cells. While each approach has its own merit, there is still much to learn about biofilm-specific regulatory networks [30] and stress resistance mechanisms, which are predicted to be related to heterogeneity [31], [32] and/or slow growth of cells [33]. Here, we used a combined approach of metabolomics and transcriptional analysis to compare extracellular matrix-embedded, wild-type *S. enterica* serovar Typhimurium (hereafter referred to as *S.* Typhimurium) to a matrix-deficient *csgD* deletion mutant. We wanted to determine if cells that lack the machinery for polymer production (due to altered regulation) would accumulate precursors and share similar metabolism as wild-type cells or whether there was a specific metabolic adaptation linked to the aggregation process. The use of luciferase reporters in transcriptional analysis allowed for temporal resolution during this early event in biofilm formation. Metabolic differentiation and stress-resistance pathways were activated in wild-type cells as part of a global transcriptional upshift coinciding with the time of aggregation. The dynamic temporal program and lack of expression in *csgD* mutant cells suggests that many of the adaptations in wild-type cells occurred in response to the microenvironment generated by aggregation. We hypothesize that growth within the self-produced matrix regulates a core set of “biofilm” traits independent of the macro environment. This could be an important step in understanding the regulation and physiology of cells in bacterial biofilms.

## Results

**Figure 1:**
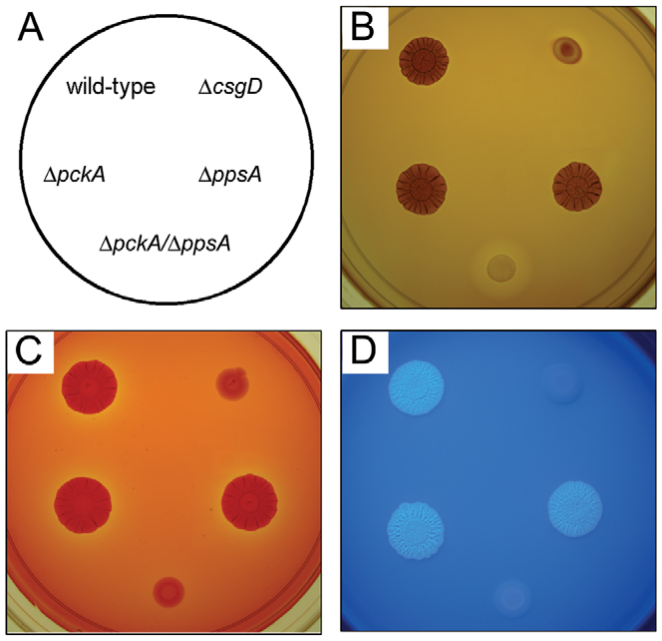
Phenotypic comparison between aggregative and non-aggregative *S.* Typhimurium strains. 1 µL of cells from overnight cultures of each strain (A) were grown at 28°C for 50 h on T agar (B), 100 h on T agar supplemented with 100 µg/mL Congo red (C), or 75 h on T agar supplemented with 200 µg/mL calcofluor (D). Colonies in (B) were stained for glycogen production (see Materials and Methods); a dark brown color is indicative of the presence of glycogen [79]. Colonies in (D) were visualized under UV light; the white color is indicative of calcofluor binding [11]. Δ*csgD* and Δ*pckA*/Δ*ppsA* strains are deficient for rdar morphotype formation (A, C), glycogen (B) and cellulose (D) production.

Characterization of the small molecule metabolites produced by bacteria represents a non-biased approach to investigate cellular activity. For our experiments, metabolites were extracted from *S.* Typhimurium wild-type and *csgD* mutant colonies grown for two or five days on 1% tryptone medium (T agar). Under these conditions, wild-type cells form aggregative, rdar morphotype colonies, whereas *csgD* deletion mutant cells form smooth colonies that lack EPS production (Figure 1; [8], [9]). Despite the differences in colony diameter (Fig. 1), the starting CFU numbers were similar: Day 2 - 2.80±0.48×10^9^ for wild-type and 1.77±0.27×10^9^ for the *csgD* mutant (n = 10, *P* = 2.8×10^−6^, two-tailed Student's paired *t*-Test); and Day 5 - 3.23±0.44×10^9^ for wild-type and 3.12±0.39×10^9^ for the *csgD* mutant (n = 8, *P* = 0.61, two-tailed Student's paired *t*-Test).

**Figure 2:**
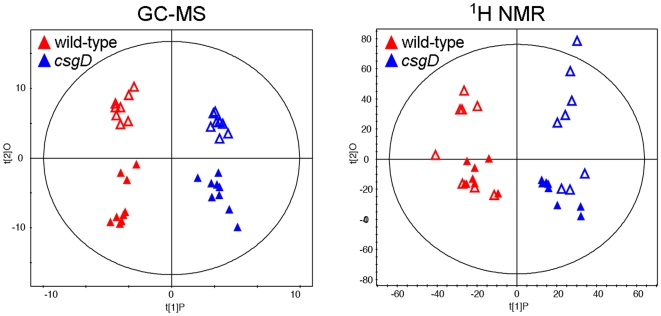
Statistical analysis and modeling of GC-MS and ^1^H NMR metabolite profiles from wild-type *S.* Typhimurium and *csgD* mutant colonies. GC-MS and ^1^H NMR profiles of metabolites extracted from wild-type or *csgD* mutant colonies grown on T agar for 2 days (open symbols) or 5 days (closed symbols) at 28°C were compared by orthogonal partial least square discriminate analysis (OPLS-DA). Score plots derived from the OPLS-DA models are shown. Each symbol represents one sample; for GC-MS, n = 9 for all groups except for *wt* at day 2 (n = 8), for ^1^H NMR, n = 8 for all four groups. The *x*-axis is the primary component, which represents all variance related to the *csgD* mutation. The *y*-axis is the first orthogonal component, which represents the variance related to the day of growth and is unrelated to mutation status.

GC-MS and ^1^H NMR metabolite profiles were initially compared by unsupervised principal component analysis [34], which confirmed that there were significant differences between strains, and the time of growth, with no sample outliers (data not shown). A final supervised model of the spectra was generated using orthogonal partial least square discriminate analysis (Figure 2). The explained variance in metabolite data (R^2^) and predictive ability (Q^2^) were high for both GC-MS (R^2^ = 0.976, Q^2^ = 0.865) and ^1^H NMR (R^2^ = 0.912, Q^2^ = 0.741) models. The GC-MS spectra were clearly divided into four groups corresponding to each strain after 2 or 5 days of growth, whereas the ^1^H NMR profiles displayed more batch variation and the groupings were not as distinct (Figure 2).

### Summary of metabolomic analysis

In total, 25 metabolites were detected at statistically different concentrations (Table 1). Many compounds detected at higher levels in wild-type colonies were the end products of gluconeogenesis, including glucose and its polymer, glycogen, as well as galactose, mannose, and glycerol-3-phosphate, an important gluconeogenesis intermediate [35]. Trehalose, glycine-betaine (betaine) and glutamate, three of the major osmoprotectants used by *S.* Typhimurium [36], were found at higher concentrations in wild-type colonies. Additional osmoprotectants detected were carnitine and galactinol (Text S1). Other compounds more abundant in wild-type colonies were glutathione, nicotinamide adenine dinucleotide (NAD^+^), octanoic acid and pyroglutamate (Text S1). The major compounds detected at higher levels in *csgD* mutant colonies were the upper TCA cycle intermediates succinate, fumarate and malate, the polyamine compounds putrescine and cadaverine, and adenosine monophosphate (AMP), adenine and proline.

**Table 1:** Metabolites with statistical difference in the wild-type *S.* Typhimurium and *csgD* mutant colonies.

Compound^a^	Concentration (µM)^d^		Fold-increase^e^	*P* value^f^
	wild-type	*csgD* mutant		
Higher in *wt* colonies				
Glucose^†^	65.4±28.2	9.47±2.35	6.9	0.0063
Trehalose^§,‡^	61.8±49.3	10.1±7.4	6.1	0.031
Glutathione^†^	34.8±13.5	9.02±6.09	3.9	0.021
Betaine^†^	11.0±5.5	3.02±2.02	3.6	0.012
Acetamide^†^	23.2±14.3	6.88±4.76	3.4	0.037
Glutamate^†^	280±133	101±31	2.8	0.034
NAD^+†^	19.4±7.1	8.47±2.08	2.3	0.018
Octanoic acid^†^	14.5±6.5	6.22±2.02	2.3	0.014
Carnitine^†^	1.49±0.38	0.63±0.58	2.4	0.001
Imidazole^†^	4.75±1.81	2.73±0.85	1.7	0.024
Glycogen^†,§,^ b	+++	+	ND	ND
Methionine^‡^	+++	+	ND	ND
Glycerol-3-phosphate^‡^	+++	+	ND	ND
Galactose^‡^	+++	+	ND	ND
Mannose^‡^	+++	+	ND	ND
Pyroglutamate^‡^	+++	+	ND	ND
Galactinol^‡,^ c	+++	+	ND	ND
Higher in Δ*csgD* colonies				
Succinate^†^	11.6±7.6	56.2±25.6	4.8	0.010
Fumarate^†,‡^	0.23±0.12	0.68±0.34	3.0	0.0066
AMP^†^	12.2±5.0	30.8±12.2	2.5	0.013
Malate^‡^	+	+++	ND	ND
Cadaverine^‡^	+	+++	ND	ND
Putrescine^‡^	+	+++	ND	ND
Proline^‡^	+	+++	ND	ND
Adenine^‡^	+	+++	ND	ND

a Individual metabolites were identified by one-dimensional^†^ or two-dimensional^§^ NMR or by GC-MS^‡^ as described in the Materials and Methods.

b Glycogen was identified at qualitatively higher levels in wild-type samples in 1D- and 2D-NMR spectrum but concentration values could not be determined.

c The common name for galactinol is 1-O-(α-D-galactopyranosyl)-myo-inositol.

d For ^1^H NMR, concentrations (± standard deviation) were determined from pooled extracts of 20 colonies (10 extracts of 2 colonies; n = 6), except for carnitine and fumarate where values are based on extracts from 2 colonies (n = 6). For GC-MS, the compounds that are listed contributed the most to the variance in the OPLS-DA model (Figure 2).

e Fold-increase represents the concentration ratio of wild-type/*csgD* mutant for compounds higher in the wild-type colonies, and *csgD* mutant/wild-type for compounds higher in *csgD* mutant colonies.

f Statistical differences between wild-type and *csgD* mutant samples were calculated using two-tailed Student's T tests assuming equal variance.

**Figure 3:**
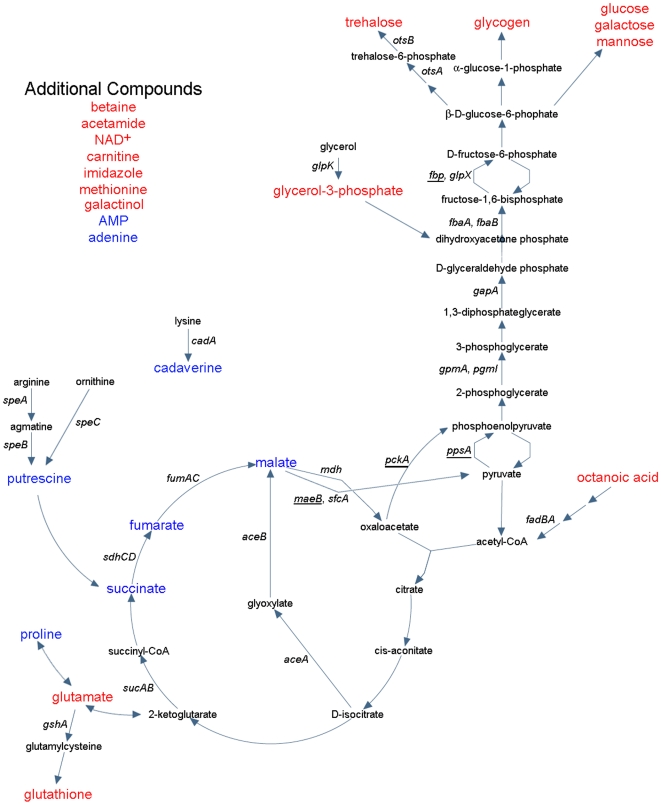
Simplified *S.* Typhimurium metabolic map displaying the results of metabolomic analaysis. Compounds shown were identified at statistically higher concentrations in wild-type colonies (red) or *csgD* mutant colonies (blue). The schematics for gluconeogenesis, the TCA cycle, and related pathways were adapted from the EcoCYC™ database (www.ecocyc.org). Genes encoding important enzymes are listed in italics; their expression was monitored using promoter luciferase fusions. Genes encoding enzymes that catalyze key reactions in gluconeogenesis are underlined.

Plotting these metabolites onto a simplified *S.* Typhimurium metabolic map (Figure 3) indicated that gluconeogenesis was specifically activated in wild-type cells and/or repressed in *csgD* mutant cells. We hypothesized that a block in gluconeogenesis in *csgD* mutant cells was responsible for the accumulation of TCA cycle intermediates and polyamine compounds. The detection of higher levels of AMP in *csgD* mutant colonies was consistent with this hypothesis, since AMP is a potent inhibitor of the gluconeogenesis-specific enzyme fructose bisphosphatase (Fbp) [37]. The presence of numerous osmoprotectants in the wild-type cells was unexpected because T agar is a low osmolarity medium; osmoprotectants normally only accumulate during growth under high osmolarity conditions [36].

### Reporters for transcriptional analysis

Based on our working model of cellular states, promoter-luciferase transcriptional fusions were generated for genes at regulatory checkpoints in several key metabolic pathways (Figure 3). In addition, reporters were generated for genes a) identified as important in related expression studies (J.S. Happe, R.J. Martinuzzi, V. Kostenko, M.G. Surette, unpublished) or b) whose protein products were identified by proteomic analysis of wild-type rdar morphotype colonies (A.P. White, W. Kim, M.G. Surette, unpublished). Control reporters that contain synthetic promoters designed to measure σ^70^ and σ^S^ activity - sig70_7 [38] and sig38H4 [9], respectively - were also included. In total, reporters were generated for 59 single gene or multiple gene operons (Table S1).

Luciferase expression was initially monitored in wild-type and *csgD* mutant strains grown on T agar as individual or mixed-strain colonies (data not shown). However, the narrow linear range of detection by the camera system made it difficult to resolve differences in expression. Furthermore, while development of the rdar colony is an ordered process, it does not occur uniformly throughout the colony [9], [39] making temporal expression profiling in the colony complicated. To overcome these problems, we analyzed gene expression during growth of the strains in 1% tryptone liquid media. Wild-type cells grown under these conditions have a clear aggregation phenotype, coupled with increased gene expression [9], and the multicellular aggregates formed share many of the characteristics of cells in rdar morphotype colonies [39], [40]. In contrast, *csgD* deletion mutant cells do not aggregate under these same growth conditions [12], [39].

### Transcriptional profiling reveals a global metabolic shift coinciding with aggregation

**Figure 4:**
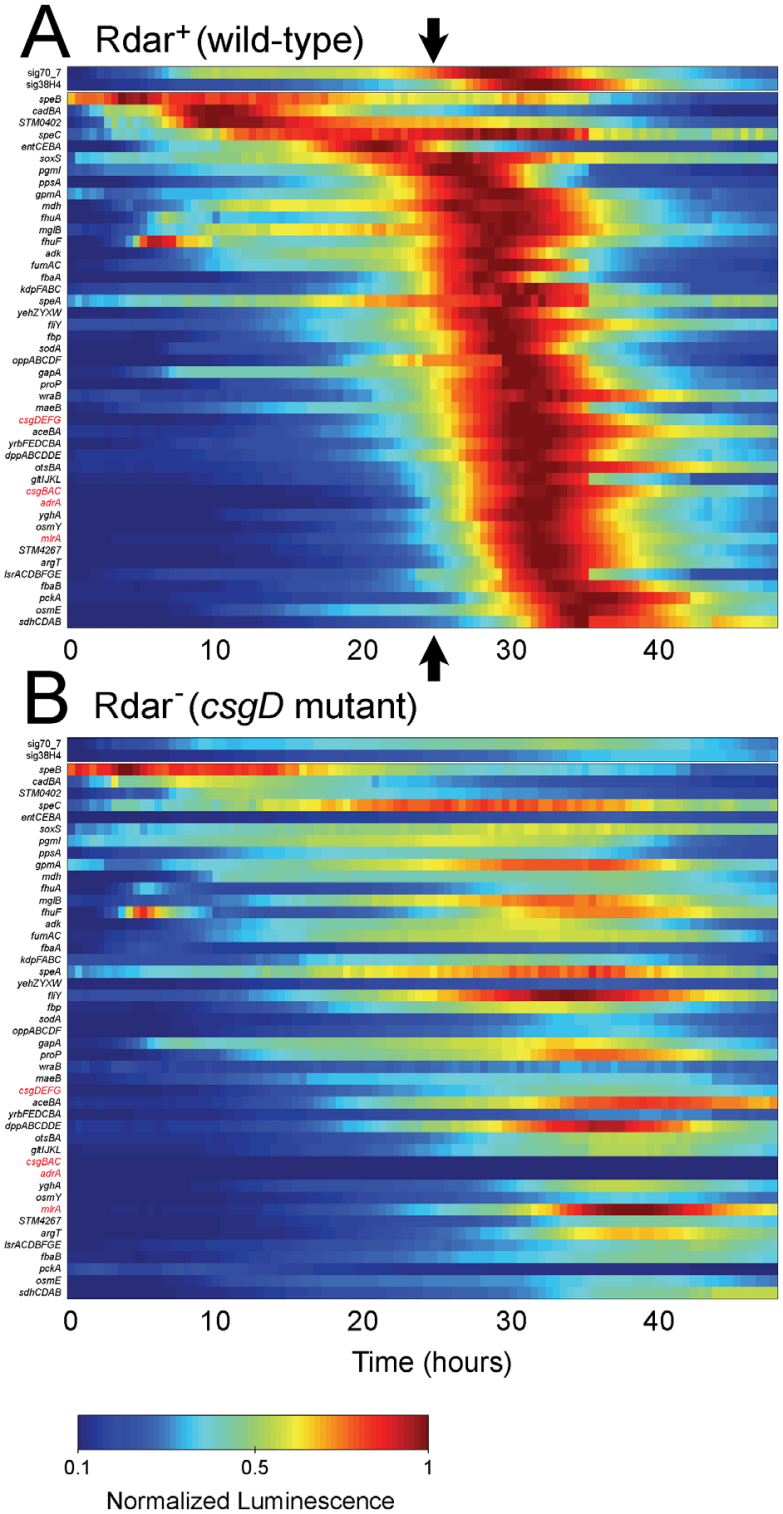
Comparison of global gene expression in aggregative (wild-type) and non-aggregative (*csgD* mutant) *S.* Typhimurium cultures during growth at 28°C. Each wild-type (A) or *csgD* mutant (B) reporter strain contains a plasmid-based promoter-luciferase (*luxCDABE*) fusion designed to measure gene expression by light production. For each reporter, shown is the ratio of *lux* activity at each time point divided by the maximum luminescence in the *wt* reporter strain. Blue and red indicate low and high expression, respectively. Gene (or operon) names are listed on the left of each panel; sig38H4 and sig70_7 are synthetic reporters designed to measure σ^S^ and σ^70^ activity, respectively. Genes that are essential for rdar morphotype formation are shown in red; *mlrA* encodes a transcriptional regulator required for *csgDEFG* expression [83]. Arrows in (A) signify the beginning of the aggregation process at 25 h.

**Figure 5:**
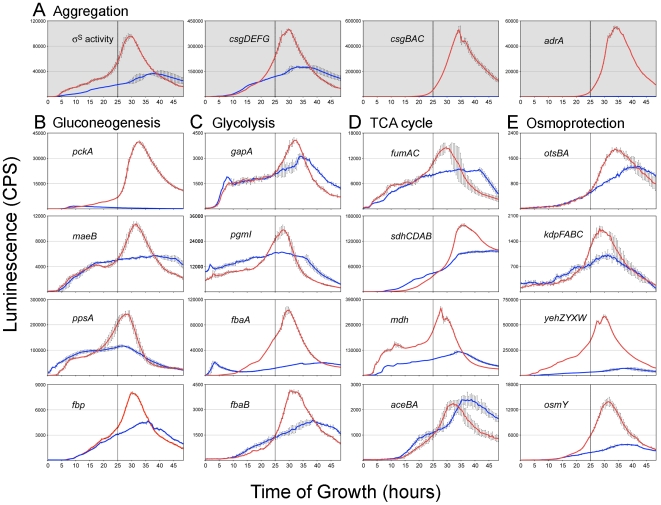
Detailed comparison of gene expression in *wt* and Δ*csgD* strains. Each red (wild-type) or blue (*csgD* mutant) curve represents the raw, non-normalized gene expression value (light counts per second; CPS) in each strain as a function of time. Expression profiles are shown for operons encoding proteins essential for rdar morphotype aggregation (A), gluconeogenesis-specific enzymes (B), glycolytic or gluconeogenic bi-directional enzymes (C), upper tricarboxylic acid cycle enzymes (D), or proteins involved in osmoprotection (E). Gene (operon) names are listed on the upper left in each panel. σ^S^ activity is represented by expression measured from the sig38H4 reporter. Vertical lines in each graph in (A) represent the beginning of the aggregation process at 25 h; these lines are shown for all other reporters to highlight the coordinated timing of gene expression. For the majority of genes, promoter activity peaks at or near the time of aggregation in wild-type cells.

Most of the reporters in *S.* Typhimurium wild-type cells displayed a distinct temporal pattern of activation with peak expression occurring at the time of aggregation (Figure 4A). Aggregation was predicted to begin at 25 h, based on an increase in σ^S^ activity and activation of essential rdar morphotype genes, including *csgDEFG*, *csgBAC* and *adrA* (Figure 5A). The coordinated activation of genes from many different functional categories (Table S1) is indicative of a global metabolic shift in wild-type cells. In contrast, the majority of operons analyzed, including the rdar morphotype genes (Figure 5A), had low expression in the *csgD* deletion mutant cultures (Figure 4B) and no correlation to the temporal pattern observed in wild-type cultures.

Global transcription rates were elevated at the time of aggregation. Wild-type cells had a two-fold increase in σ^70^ activity and nearly three-fold increase in σ^S^ activity relative to *csgD* mutant cells (Figure 4, Table S1). Since σ^70^ and σ^S^ compete for binding to the RNA polymerase holoenzyme and drive expression of genes required for vegetative growth and stress responses, respectively [14], [15], these results were indicative of physiological differences between wild-type and *csgD* mutant cultures.

### Carbon flux into gluconeogenesis is increased in *S.* Typhimurium rdar morphotype cells

To monitor carbon flux, we analyzed the expression of genes encoding key enzymes in gluconeogenesis, glycolysis and TCA cycles (Figure 3). Four key gluconeogenesis-specific enzymes, malic enzyme (*maeB*), PEP synthase (*ppsA*), PEP carboxylase (*pckA*) and fructose bisphosphatase (*fbp*) [35], were all significantly up-regulated in wild-type cultures relative to *csgD* mutant cultures (Figure 4, Figure 5B). The largest change in gene expression was measured for *pckA*, which was elevated 45-fold (Table S1). *GpmA*, *pgmI*, *gapA*, *fbaA*, and *fbaB* genes, encoding enzymes that catalyze reversible steps in gluconeogenesis and glycolysis, were also induced in the wild-type strain at the time of aggregation (Figure 4A, Figure 5C). We hypothesized that elevated expression of these enzymes was necessary for increased carbon flux between PEP and fructose-1,6,-bisphosphate (Figure 3). Since upper TCA cycle intermediates are essential starting points for gluconeogenesis [41], we monitored the expression of succinate dehydrogenase (*sdhCD*), fumarate reductase (*fumAC*), and malate dehydrogenase (*mdh*); each of these enzymes were up-regulated in wild-type cultures coinciding with the time of aggregation (Figure 4A, Figure 5D). Lower expression of these genes in the *csgD* mutant cultures indicated that the elevated concentrations of TCA intermediates measured by metabolomics were not due to increased amounts of enzyme, but were more likely caused by inhibition of gluconeogenesis. Expression of *aceBA*, coding for enzymes in the glyoxylate shunt, and *sucAB*, coding for enzymes catalyzing conversion of 2-ketoglutarate to succinate for complete TCA cycling, were not different between wild-type and *csgD* mutant strains (Figure 4, Figure 5D, Table S1). For the majority of metabolic reporters analyzed, expression profiles were similar in both strains until the estimated time of aggregation at which point expression was induced in wild-type cells (Figure 5B, C, D). This confirmed that there was a metabolic shift linked to the aggregation process.

The production of sugars from gluconeogenesis should be an essential pathway for aggregation in *S.* Typhimurium, particularly when strains are grown on amino-acid based media, such as tryptone or LB [42]. This was confirmed here since a *ppsA*/*pckA* mutant strain was unable to form rdar morphotype colonies and synthesize EPS or glycogen (Figure 1). However, strains carrying single deletions in *ppsA* or *pckA* were not impaired indicating that either arm of gluconeogenesis was sufficient to generate the precursor sugars required for polysaccharide production. Since gluconeogenesis is an energy consuming process that may be controlled by the adenylate energy charge [43], we also investigated the expression of adenylate kinase (Adk). Adk catalyzes the reversible conversion of ATP+AMP into two ADP molecules and is known to buffer ATP levels during periods of rapid ATP consumption [44]. *Adk* expression was elevated in wild-type cultures relative to *csgD* mutant cultures (Figure 4; Table S1), suggesting that aggregating cells have an increased requirement for ATP. Furthermore, since the Adk reaction is the only route of de novo synthesis of ADP from AMP in *Salmonella* [44] these results could explain the increased AMP levels detected in *csgD* mutant colonies.

### Enzymes for osmoprotectant synthesis and accumulation are up-regulated in *S.* Typhimurium rdar morphotype cells

*OtsBA*, coding for enzymes that catalyze trehalose biosynthesis [36], and *kdpFABC*, which encodes a high affinity potassium import system coupled to glutamate accumulation [36] were up-regulated in wild-type cultures at the time of aggregation (Figure 5E). Glycine betaine (betaine) or its precursor, choline, cannot be synthesized de novo by *Salmonella* [44] but can be transported into cells via the well-characterized *proP* and *proU* (*proVWX*) import systems [36]. Expression of *proP* was induced in the wild-type cultures (Figure 4), but *proVWX* was not (Table S1). Proline can also act as an osmoprotectant and be transported through the *proP* and *proU* systems [36], however, we could not explain the higher levels of proline in *csgD* mutant colonies based on these results. *yehZYXW*, encoding a putative osmoprotectant import system [45], and *osmE* and *osmY*, osmotically-inducible genes encoding proteins of unknown function [14], were highly induced in wild-type cultures timed with aggregation (Figure 4, Figure 5E, Table S1). This suggested that during the aggregation process, cells in wild-type cultures were exposed to an environment of increased osmolarity.

### Defences against reactive oxygen species (ROS) are induced in *S.* Typhimurium rdar morphotype cells

The tri-peptide glutathione (L-γ-glutamylcysteinylglycine; GSH) is a major reducing agent and acts as a detoxifying compound through non-enzymatic deactivation of ROS and the action of glutathione-S-transferase enzymes [46]. Expression of *gshA*, coding for the enzyme catalyzing the first step in GSH synthesis, was not elevated in the wild-type background (Table S1). However, *STM4267*, encoding a glutathione-S-transferase, and *yghA*, encoding a putative glutathionylspermidine synthase, were up-regulated in wild-type cells coinciding with aggregation (Figure 4, Table S1). Glutathionylspermidine, a conjugate of GSH and spermidine, can also function as a detoxifying compound [47]. Several oxidative stress-relieving enzymes, including cytosolic superoxide dismutase (SodA), a putative peroxidase (STM0402), and a NADH∶quinone oxidoreductase (WrbA) [48] were identified as abundant by proteomic analysis of wild-type rdar morphotype colonies (data not shown). Each of these genes, along with *soxS* from the *soxRS* superoxide response regulon [49], were expressed at higher levels in wild-type cultures (Figure 4, Table S1). Increased *wraB* expression in wild-type cells could explain the increased levels of oxidized NAD^+^ detected by metabolomics.

### Expression levels of polyamine biosynthesis enzymes are similar in *wild-type S.* Typhimurium and *csgD* mutant strains

Polyamines have diverse roles within cells, including stabilization of phosphate charges on nucleic acids and other negatively charged polymers and scavenging of ROS [50]. We analyzed expression of *speA*, *speB*, *speC*, and *cadBA* genes encoding four of the main decarboxylation enzymes for production of putrescine and cadaverine (Figure 3). *speA*, *speB* and *speC* had similar magnitudes of expression in wild-type and *csgD* mutant cultures, whereas *cadBA* was slightly elevated in wild-type cultures (Figure 4, Table S1). These results fit our hypothesis that polyamines accumulated in *csgD* mutant cells as a result of reduced carbon flux into gluconeogenesis and other biosynthetic pathways.

### Levels of intracellular iron are limiting during growth in 1% tryptone

Iron limitation is known to activate *csgD* expression and formation of the rdar morphotype [8] and is also known to induce expression of different iron acquisition systems [51]. *EntCEBA*, encoding enzymes for the biosynthesis of enterobactin, *fhuA*, encoding an outer membrane receptor for ferrichrome siderophores produced by fungi, and *fhuF*, which encodes a protein involved in the ferrioxamine B system [52], were induced in both wild-type and *csgD* mutant cultures during growth (Figure 4), indicating that iron was limiting during growth. However, the *fhuA* and *entCEBA* operons were induced higher in wild-type cultures (Table S1), suggesting that aggregation may also affect intracellular iron levels.

### ABC transporters are up-regulated during gro*wild-type*h

In Gram-negative bacteria, many transporters from the ATP-binding cassette (ABC) superfamily function as nutrient importers that utilize high-affinity periplasmic-binding proteins (PBP) to define their specificity [53]. Several PBP, with specificities for carbohydrates, amino acids, peptides, or unknown substrates (Table S1), were identified as abundant by proteomic analysis of wild-type rdar morphotype colonies (data not shown). Expression of these operons, including *lsrACDBFGE*, encoding the transport and processing system for the AI-2 signalling molecule [54], were induced in both strain backgrounds at later time points during growth when nutrient limitation would occur (Figure 4, data not shown). *Yrb*, *opp*, *lsr*, *glt* and *argT* operons were up-regulated in wild-type cultures coinciding with aggregation (Figure 4; Table S1). The induction of diverse nutrient import systems may be necessary for cells to harvest all available nutrients in the current growth media or could represent an example of carbon source foraging [55], where cells expend energy to broaden their search for alternative energy sources.

## Discussion

Starvation in non-differentiating bacteria is known to induce a myriad of molecular changes to allow for more efficient nutrient scavenging and increased stress resistance [56]. The results described indicate that the *Salmonella* rdar morphotype is a specialized multicellular physiology adapted to this survival response. This may be critical for *Salmonella* transmission by ensuring that enough cells survive to infect new hosts [9], [13], [21]. Analyzing the metabolome and identification of the major metabolites by NMR and GC-MS revealed that rdar morphotype cells have a shift in central metabolism to gluconeogenesis and production of small molecules that aid in osmotic stress response. These changes were observed at the transcriptional level as part of a global temporal shift that was timed with aggregation.

*S.* Typhimurium rdar morphotype cells displayed increased carbon flux into gluconeogenesis at the onset of aggregation. In particular, PEP synthase and PEP carboxylase enzymes were required to synthesize sugars for production of EPS and glycogen. This result was undoubtedly influenced by growth on amino-acid based media, however, the significant up-regulation of gluconeogenesis in aggregation-positive wild-type cells compared to aggregation-negative *csgD* mutant cells was striking. This observation has implications for many types of natural biofilms and is likely not restricted to *Salmonella*. Polysaccharides are usually essential for aggregation to occur [6], [11], such as with alginate, Pel and Psl polysaccharides in *Pseudomonas aeruginosa* [57], and VPS in *Vibrio cholerae* [58]. Glycogen is also important because it is known to enhance *S. enterica* survival [59] and was recently shown to play a critical role in transmission of *V. cholerae* [60]. Under the conditions investigated, it is assumed that carbon flux is controlled by the catabolite repressor/activator (Cra) protein, which activates gluconeogenesis enzymes (*ppsA*, *pckA*, *fbp*) and represses sugar catabolism enzymes [61]. In agreement with this hypothesis, the addition of glucose during growth leads to inhibition of the rdar morphotype (A.P. White and M.G. Surette, unpublished). Collectively, these results suggest that blocking gluconeogenesis may be an effective means to prevent or reduce biofilm formation in a wide variety of bacteria.

*S.* Typhimurium rdar morphotype cells displayed numerous stress-resistance adaptations that coincided with aggregation. Several osmoprotectants were detected at high levels in rdar morphotype colonies and transcriptional analysis verified that systems for osmoprotectant synthesis and transport were induced. Osmoprotectants are predicted to enhance desiccation survival by causing a reduction in water stress [62]. We also observed that wild-type cells had an increased capacity for ROS defence, which would partially alleviate the damage caused to DNA, lipids and proteins known to occur during desiccation [62]. Finally, the induction of nutrient acquisition systems as part of a carbon foraging or starvation response [55], [56] would ensure swift revival of cells after long periods of “metabolic dormancy”. Our results agree with a recent study by Hinton and colleagues [63] who investigated *S.* Typhimurium biofilms using proteomic and microarray analysis. Similar stress-resistance adaptations have also been observed in other biofilm systems, including evidence for increased osmoprotection in *E. coli* [27] and ROS defence in *P. aeruginosa* [28]. Each of these main stress adaptations are known to be controlled by σ^S^ [14], [15], [55], [56], and σ^S^ activity was measured to be almost three times higher in *S.* Typhimurium wild-type cells compared to *csgD* mutant cells. In *E. coli*, which shares most features of rdar morphotype regulation [17], CsgD was shown to have a stabilizing effect on σ^S^ protein levels [64] which could partially explain our findings. The only other metabolome comparison of biofilm and planktonic cells was performed with *P. aeruginosa*, and although these cell types had different metabolic profiles, individual metabolites were not identified [65].

One of the most intriguing questions arising from this study is how is the signal for *Salmonella* aggregation linked to metabolism and stress resistance? The transcriptional regulator CsgD is the most obvious candidate, acting in concert with σ^S^ [63], [64]. However, analysis of the CsgD regulon in *E. coli*, did not reveal any gene targets linked to global carbon flux and relatively few that were directly linked to stress resistance [66]. Based on these findings, we hypothesize that the primary role of CsgD is to control the aggregation process and that the majority of adaptations are the consequence of production of an extracellular matrix. Stress-inducing changes in the microenvironment of aggregated or biofilm cells have been observed before. In *P. aeruginosa*, the chelation of ions by extracellular DNA present in the biofilm caused activation of antibiotic and stress resistance pathways in the adjacent cells [5]. It is possible that synthesis of an extracellular matrix by rdar morphotype cells causes an increase in the local osmolarity around aggregated cells [6] or mimics an increase in osmolarity by reducing the water activity, which, in turn, would expose cells to increased oxidative stress [62]. The signal for *S.* Typhimurium cells to aggregate and the accompanying changes may be akin to a developmental process, such as sporulation in *Myxococcus* and *Bacillus* spp. [67], [68]. Another possibility is that the rdar morphotype adaptations are genetically programmed changes that occur prior to experiencing the environmental stress (anticipatory regulation [69]).

c-di-GMP is a key regulatory molecule in the aggregation process. High intracellular levels have been linked to aggregation in numerous bacterial species, including *Salmonella*, *E. coli*, *Pseudomonas spp.* and *Vibrio spp.* [3], [70]. In *Salmonella* and *E. coli*, there is a complex interplay between c-di-GMP, CsgD, σ^S^, and other global regulators, such as CsrA (Text S1) [16], [17], [71]. Although we didn't attempt to dissect this regulatory network, both curli production (via activation of *csgD*) and cellulose production (via activation of *adrA*) are indicators of high intracellular levels of c-di-GMP [16]. While cellulose production through AdrA appears to be a very specific response to a specific c-di-GMP signalling pathway, a recent study challenges the role of some diguanylate cyclases in modulating cytoplasmic c-di-GMP pools in *S.* Typhimurium [72]. There is evidence that a high concentration of c-di-GMP can regulate expression of *soxS* (ROS defence), *fur* (iron acquisition), and other global regulatory proteins in *E. coli* [73], however the physiological relevance of this study is questionable. In recent experiments performed with *P. aeruginosa*, Starkey et al. [74] found that the number of genes regulated in response of c-di-GMP was relatively small compared to the number of genes differentially regulated as a result of aggregation.

**Figure 6:**
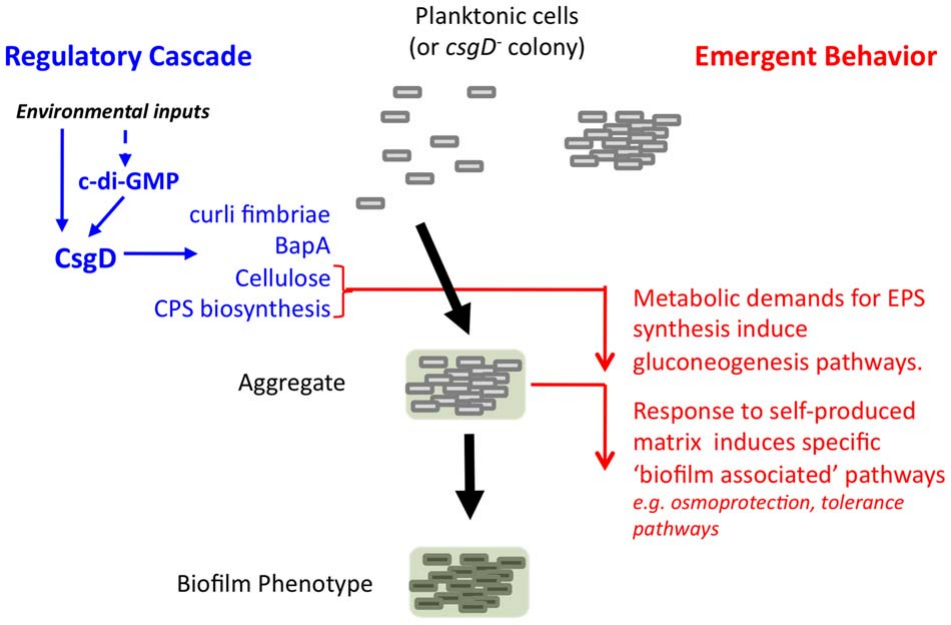
Model of biofilm development dependent on cellular response to self-produced extracellular matrix. Aggregation is initiated by the activation of the CsgD regulon; BapA is a large cell-surface protein involved in biofilm formation [84]. The metabolic demand of polysaccharide production leads to induction of gluconeogenesis and the subsequent response to the self-produced matrix activates pathways that lead to general biofilm phenotypes. These later processes represent emergent behaviors and are not under control of a “biofilm specific’ regulatory cascade.

The elaborate temporal program associated with the *Salmonella* rdar morphotype is initiated through the aggregation regulator CsgD. The demands for exopolysaccharide production in turn cause changes in the expression of metabolic genes associated with gluconeogenesis. Finally the microenvironment that results from being embedded in a self-produced matrix results in the induction of numerous pathways associated with stress tolerance. Thus what appears to be a defined temporal program is not coordinated through a master regulatory pathway but is the result of the cell producing and responding to its own matrix (Fig. 6). Since growth in multicellular aggregates and biofilms is common among microorganisms, our findings may represent a general phenomenon that helps to explain some of the inherent resistant properties of biofilms.

## Materials and Methods

### Bacterial strains, media and growth conditions

*S.* Typhimurium strain ATCC 14028 was used as the wild-type strain in this study. The isogenic *csgD* mutant strain (Δ*csgD*), previously named Δ*agfD* [9], has a 612 bp deletion in *csgD* (encoding amino acids 6 to 210 in the mature protein). Strains were grown for 16–20 h at 37°C with agitation in Miller's Luria-Bertani broth (1.0% salt) or LB without salt (LBns), supplemented with 50 µg/ml Kanamycin (Kan), if necessary, before performing additional experiments. To obtain colonies, 1 µl aliquots of overnight cultures were spotted on T agar (1% tryptone, 1.5% Difco agar) and incubated at 28°C for up to 5 days. For bioluminescence assays, reporter strain cultures were diluted 1 in 600 in T broth supplemented with 50 µg/ml Kan to a final volume of 150 µl in 96-well clear-bottom black plates (9520 Costar; Corning Inc.). The culture in each well was overlaid with 50 µl mineral oil prior to starting the assays. Cultures were assayed for luminescence (0.1s) and absorbance (620 nm, 0.1s) every 30 min during growth at 28°C with agitation in a Wallac Victor^2^ (Perkin-Elmer Life Sciences, Boston, Mass.).

### Construction of luciferase reporters

Promoter-containing DNA regions were PCR amplified from wild-type *S.* Typhimurium, purified (Qiagen Inc.), digested with *Xho*I and *Bam*HI (Invitrogen Canada Inc.), and ligated using T4 DNA ligase (Invitrogen Canada Inc.) into pCS26-Pac (*Xho*I-*Bam*HI) or pU220 (*Bam*HI-*Xho*I) reporter vectors containing the *luxCDABE* operon from *Photorhabdus luminescens* [75]. All primers used for reporter construction are listed in Table S2. *Salmonella* strains were transformed with plasmids via electroporation (Bio-Rad Laboratories Inc.).

The *csgDEFG (agfDEFG)*, *csgBAC (agfBAC)*, *adrA* and *mlrA* reporters have been previously described [9], [22]. The promoter sequences in the sig38H4 [9] and sig70_7 [38] reporters are (ATAATTCCATGCGGTTTCGCTAAAATCATGTATACTTATTATCAATT) and (AATAATTCTTGATATTTATGCTTCCGGCTCGTATTTTACGTGCAATT), respectively; the −35 and −10 promoter regions are underlined. These reporters were selected from a library constructed with the above sequences with four degenerate positions in each promoter (K. Pabbaraju and M.G. Surette, unpublished). Light production as the result of transcription from these synthetic promoters reflects σ^S^- or σ^70^-RNA polymerase activity.

### Construction and characterization of *S.* Typhimurium deletion *mutants*

Δ*ppsA* and Δ*pckA* mutant strains were created by deletion mutagenesis of wild-type *S.* Typhimurium using a chloramphenicol cassette as described [76]. Chromosomal loci of the generated mutants were verified by PCR using a primer specific to the insert and a primer that annealed to sequence that flanked the disrupted loci (Table S2). To ensure the absence of secondary mutations, all generated deletions were moved into a clean wild-type background by P22 transduction [77]. The *pckA::cat* mutant was cured of the chloramphenicol cassette as previously described [76]. The unmarked Δ*pckA* mutant was transduced with the P22 lysate of *ppsA::cat* to generate a Δ*pckA*/Δ*ppsA* double mutant. The mutants were phenotypically tested by examining their capacity to grow in M9 minimal media supplemented with 0.2% glucose, 0.2% glycerol, 0.4% acetate, 0.4% citrate, or 0.4% succinate as previously described [78].

### Staining of colonies for glycogen production

An aqueous iodine solution (0.01 M I_2_, 0.03 M KI) [79] was initially tested for glycogen staining but did not stain glycogen intensely enough. Therefore, the iodine concentration was increased to 0.1 M and the solution was vortexed for 5 min prior to staining. 5 mL of solution was added to each plate and swirled around the entire plate area and left to stain for 5 min before taking pictures.

### Extraction of metabolites from *S.* Typhimurium colonies

Wild-type or *csgD* mutant colonies were removed from T agar after 2 or 5 days growth and placed into 2 mL sterile vials containing 0.2 g of 0.1 mm Zirconia/Silica beads (BioSpec Products Inc., Bartlesville, OK, USA); two colonies were added to each vial. Immediately following the addition of 1 mL of ice-cold methanol, cells were homogenized for 2 min using a Mini-Beadbeater 8 (BioSpec Products Inc., Bartlesville, OK, USA). Beads and cell debris were sedimented by centrifugation (20,000g, 2 min), the supernatant was removed and filtered through a 0.22 µ Spin-X centrifuge tube filter (Costar, Corning Inc) by centrifugation (20,000g, 1 min). Samples were evaporated to dryness using a Centrivap concentrator (Labconco Corp., Kansas City, MO) and were stored at −80°C prior to NMR or GC-MS analysis. We chose to extract metabolites using ice-cold methanol because this method yielded the most comprehensive array of metabolites in *E. coli* when six commonly used procedures were compared [80]. To determine the number of colony forming units (CFU) at the time of extraction, colonies removed from agar were resuspended in 0.5 mL of phosphate-buffered saline, homogenized in a tissue grinder for ∼20 s, serially diluted in triplicate, plated in duplicate in 5 µL drops onto LB agar and incubated at 28°C overnight.

### Preparation of samples for ^1^H NMR analysis

Dried samples were resuspended in 600 µL of deionized water and filtered through pre-wetted NanoSep 3K filters (Pall, Ann Arbor, MI, USA) by centrifugation (20,000g, 60min) to remove any dissolved proteins. 130 µL of metabolite sample buffer (0.5 M sodium phosphate (monobasic)+2.5 mM 2,2-dimethyl-2-silapentane-5-sulfonate (DSS)) and 10 µL of 1M sodium azide was added to bring the volume of each sample to ∼650 µL. pH values ranged between 7.2 and 7.4 for all samples tested (data not shown), therefore samples were not pH-adjusted prior to analysis.

### Preparation of samples for GC-MS analysis

For GC-MS, dried samples were resuspended in 60 µl of methoxyamine in anhydrous pyridine (20 mg/ml), transferred to a glass vial and incubated overnight at room temperature on a rotary shaker. 60 µl of *N*-methyl-*N*-trimethylsilyltrifluoroacetamide (MSTFA) and 6.0 µl of chlorotrimethylsilane (TMS-Cl) were added and the reaction was continued for one hour. A 100 µl aliquot of the reaction mixture was diluted with 900 µl of hexane prior to analysis.

### ^1^H NMR analysis

All experiments were performed on a Bruker Advance 600 MHz spectrometer (Bruker Daltonics) operating at 600.22 MHz and equipped with a 5-mm TXI probe at 298 K for solution-state analysis. All one-dimensional ^1^H NMR spectra were acquired using a standard Bruker noesypr1d pulse sequence in which the residual water peak was irradiated during the relaxation delay of 1.0 s and during the mixing time of 100 ms. A total of 256 scans were collected into 65,536 data points over a spectral width of 12,195 Hz, with a 5-s repetition time. A line broadening of 0.5 Hz was applied to the spectra prior to Fourier transformation, phasing and baseline correction. To confirm spectral assignments, a ^1^H,^13^C heteronuclear single quantum correlation (HSQC) and a ^1^H,^1^H total correlation (TOCSY) spectra were acquired. A standard echo/antiecho-TPPI gradient selection pulse sequence [81] was used for HSQC spectrum. The parameters comprised a J-coupling delay of 0.86 ms, time domain points of 2 k (F2) and 256 (F1), spectral width (^1^H) of 12 ppm, spectral width (^13^C) of 169 ppm, GARP ^13^C decoupling, 80 scans/increment, acquisition time of 0.14 s, and a relaxation delay of 1.6 s. A phase sensitive homonuclear Hartman-Hahn transfer using DIPSI2 sequence for mixing with water suppression using exitation sculping with gradients [82] was used for TOCSY spectrum with parameters comprised a TOCSY mixing time 0.12 s, time domain points of 2k (F2) and 400 (F1), spectral width (both ^1^H) of 12 ppm, 64 scans/increment, acquisition time of 0.14 s, and a relaxation delay of 1.0 s.

Metabolite identification and quantification from one-dimensional ^1^H NMR spectra was achieved using the Profiler module of Chenomx NMR Suite version 4.6 (Chenomx. Inc., Edmonton, Canada). Chenomx Profiler is linked to a database of metabolites whose unique NMR spectral signatures are encoded at various spectrophotometer frequencies, including 600 MHz. Two-dimensional ^1^H NMR was employed to confirm compound identities where necessary. Metabolites were quantified by comparison to the internal standard DSS, which also served as a chemical shift reference.

### GC-MS analysis

Experiments were performed on an Agilent 5975B inert XL gas chromatograph (6890N) and mass spectrometer (EI/CI) (Agilent Technologies Canada Inc., Mississauga, Ont). Individual metabolites were identified by comparison to the HSALLMASS compound database using the Agilent MSD Security ChemStation software.

### Chemometric Analysis

One-dimensional ^1^H NMR spectra were imported into Chenomx NMR Suite version 4.6 (Chenomx) for spectral binning. All shifts related to the solvent (i.e., in the range of 4.5–5.0 ppm) and DSS were excluded, and the remaining spectral regions were divided into 0.04-ppm bins. GC-MS spectra were also divided into 0.04-ppm bins. GC-MS spectra were processed as peaks deconvoluted using the Automated Mass Spectral Deconvolution and Identification System (AMDIS, Version 2.64, NIST, US) and subsequently filtered using spectconnect (http://spectconnect.mit.edu/; PMID: 17263323). Chemometric analysis was performed using SIMCA-P version 11.5 (Umetrics) with unsupervised PCA (to look for outliers and other anomalous sources of variance) or orthogonal partial least square discriminate analysis (OPLS-DA). Variables were scaled to unit variance to ensure equal contributions to the models.

### Statistical Analysis

Statistical differences in metabolite concentrations or reporter gene expression (maximum CPS values) between the wild-type and *csgD* mutant strains were calculated using Student's paired *t*-Tests, with a two-tailed distribution.

## Supporting Information

Table S1 Comparison of promoter-luciferase reporter expression in wild-type *S.* Typhimurium and *csgD* deletion mutant strains.
(0.21 MB DOC)

Table S2 PCR primers used for reporter construction or mutagenesis.
(0.21 MB DOC)

Text S1 (0.12 MB DOC)
