# Supplementary material for: A Global Metabolic Shift Is Linked to Salmonella Multicellular Development
Source: PLoS One. 2010 Jul 27;5(7):e11814. doi: 10.1371/journal.pone.0011814 (PMC2910731; doi:10.1371/journal.pone.0011814)
Supplement: Table S1 — Comparison of promoter-luciferase reporter expression in wild-type S. Typhimurium and csgD deletion mutant strains. (0.21 MB DOC) [file pone.0011814.s001.doc]

**Table S1. Comparison of promoter-luciferase reporter expression in wild-type *S.* Typhimurium and *csgD* deletion mutant strains.**

| Pathway or Process  *lux reporter*^a^ | Description^b^ | Fold-increase ^c^ | *P* value^d^ |
| --- | --- | --- | --- |
|  |  |  |  |
| Aggregation; Rdar morphotype |  |  |  |
|  |  |  |  |
| *csgBAC*^†^ | Curli (Tafi) structural subunits | 1340 ± 680 | 5.4 × 10^-7^ |
| *adrA*^†^ | GGDEF-enzyme, generates c-di-GMP required for cellulose biosynthesis | 348 ± 87 | 0.0071 |
| *csgDEFG*^†^ | curli assembly; CsgD acts as a transcriptional activator | 2.2 ± 0.6 | 7.1 × 10^-8^ |
| *mlrA*^†^ | MerR-like transcriptional regulator; involved in *csgD* activation | 1.2 ± 0.6 | 0.64 |
| Transcription; Sigma factor activity |  |  |  |
|  |  |  |  |
| *sig38H4*^†^ | Synthetic RpoS(σ^38^)-dependent promoter; RpoS activity | 2.9 ± 0.5 | 4.3 × 10^-5^ |
| *sig70_7*^†^ | Synthetic RpoD(σ^70^)-dependent promoter; σ^70^ activity | 2.0 ± 0.3 | 0.013 |
| TCA, Glycolysis, and Gluconeogenesis (gluconeogenesis) enzymes |  |  |  |
|  |  |  |  |
| *pckA*^§^ | Phosphoenolpyruvate carboxykinase | 46 ± 22 | 0.029 |
| *fbaA*^§^ | Fructose bisphosphate aldolase II | 4.3 ± 1.3 | 0.0050 |
| *sdhCDAB*^§^ | Succinate dehydrogenase complex | 3.3 ± 1.0 | 0.0049 |
| *mdh*^§,‡^ | Malate dehydrogenase | 2.6 ± 1.1 | 9.2 × 10^-5^ |
| *maeB*^§^ | Malic enzyme, NADP-dependent | 2.2 ± 0.4 | 0.0097 |
| *ppsA*^§^ | Phosphoenolpyruvate synthase | 2.1 ± 0.5 | 0.0021 |
| *fbaB*^†^ | Fructose bisphosphate aldolase I | 2.0 ± 0.3 | 0.0010 |
| *fumAC*^§^ | Fumarate reductase A and C | 1.7 ± 0.3 | 0.0013 |
| *fbp*^†^ | Fructose bisphosphatase | 1.5 ± 0.2 | 3.0 × 10^-6^ |
| *pgmI*^†^ | Phoshoglyceromutase, bisphosphoglycerate independent | 1.5 ± 0.3 | 0.00010 |
| *gapA*^§^ | Glyceraldehyde-3-phosphate dehydrogenase A | 1.4 ± 0.1 | 3.9 × 10^-6^ |
| *gpmA*^†^ | Phosphoglyceromutase, bisphosphoglycerate dependent | 1.4 ± 0.3 | 0.016 |
| *aceBA*^†^ | Glyoxylate shunt enzymes | 1.4 ± 0.7 | 0.52 |
| *sucAB*^†^ | 2-Oxoglutarate dehydrogenase complex | 1.2 ± 0.2 | 0.12 |
| *glpFKX*^†^*,*^‡^ | Glycerol conversion into glycolysis | 0.8 ± 0.6 | 0.33 |
| Osmoprotection |  |  |  |
|  |  |  |  |
| *yehZYXW*^§^ | Osmoprotectant transport system | 6.1 ± 3.2 | 0.019 |
| *osmY*^†,‡^ | Osmotically-inducible periplasmic protein | 2.7 ± 0.9 | 0.028 |
| *osmE*^§^ | Osmotically-inducible lipoprotein | 2.5 ± 0.4 | 0.00065 |
| *kdpFABC*^†^ | High-affinity potassium transporter, ATP-dependent | 2.0 ± 0.3 | 0.0042 |
| *otsBA*^†^ | Trehalose synthase | 1.7 ± 0.5 | 0.013 |
| *proP*^§^ | Proline, betaine transporter | 1.5 ± 0.4 | 0.089 |
| *proVWX*^†^ | ProU import system | 0.8 ± 0.6 | 0.16 |
| Detoxification of reactive oxygen species |  |  |  |
|  |  |  |  |
| *wraB*^†,‡^ | NADH:quinone oxidoreductase | 3.1 ± 0.5 | 0.0030 |
| *STM4267*^§^ | Putative glutathione S-transferase | 2.9 ± 1.4 | 0.0020 |
| *yghA*^†,‡^ | Putative glutathionyl spermidine synthase | 1.9 ± 0.4 | 0.0033 |
| *sodA*^†^*,*^‡^ | Mn-dependent superoxide dismutase | 1.8 ± 0.3 | 0.012 |
| *STM0402*^†,‡^ | Putative thiol alkyl hydroperoxide reductase | 1.8 ± 0.5 | 0.0081 |
| *soxS*^†^ | Superoxide response; transcriptional regulator | 1.5 ± 0.3 | 0.68 |
| *gshA*^†^ | Glutamyl-cysteinyl ligase (GSH synthase) | 1.3 ± 0.9 | 0.14 |
| Putrescine/Cadaverine synthesis |  |  |  |
|  |  |  |  |
| *cadBA*^†^ | Cadaverine/lysine antiporter; lysine decarboxylase | 1.3 ± 0.1 | 0.0074 |
| *speA*^†^ | Arginine decarboxylase | 1.3 ± 0.4 | 0.37 |
| *speC*^†^ | Ornithine decarboxylase | 1.2 ± 0.3 | 0.30 |
| *speB*^†^ | Agmatinase | 0.8 ± 0.1 | 0.051 |
| Iron acquisition |  |  |  |
|  |  |  |  |
| *entCEBA*^§^ | Enzymes involved in synthesis of enterobactin | 4.9 ± 0.5 | 0.00013 |
| *fhuA*^§^ | Outer membrane receptor for ferrichrome | 2.3 ± 0.5 | 0.0085 |
| *fhuF*^§^ | Ferric hydroxamate transport, involved in reduction of ferric iron in cytoplasmic ferrioxamine B | 1.1 ± 0.1 | 0.025 |
| ABC transporter systems; specificities are shown |  |  |  |
|  |  |  |  |
| *yrbFEDCB*^†,‡^ | Unknown substrate(s) | 6.4 ± 4.0 | 0.029 |
| *oppABCDF*^†,‡^ | Oligopeptides | 3.4 ± 0.7 | 0.00037 |
| *lsrACDBFGE*^†^ | Auto-inducer 2 | 2.4 ± 0.6 | 0.0020 |
| *gltIJKL*^†,‡^ | Glutamate | 2.2 ± 0.7 | 0.00053 |
| *argT*^†,‡^ | Lysine, Arginine, Ornithine | 1.5 ± 0.3 | 0.072 |
| *mglBAC*^†,‡^ | Galactose, glucose | 1.3 ± 0.3 | 0.11 |
| *ugpBAECQ*^†^*,*^‡^ | Glycerol-3-phosphate | 1.3 ± 0.7 | 0.60 |
| *fliY*^†,‡^ | Cystine | 1.2 ± 0.3 | 0.88 |
| *dppABCDE*^†,‡^ | Dipeptides | 1.2 ± 0.6 | 0.41 |
| *rbsDACBKR*^†^*,*^‡^ | Ribose | 0.9 ± 0.3 | 0.52 |
| Glycine |  |  |  |
|  |  |  |  |
| *gcvTHP*^†,‡^ | Glycine cleavage complex | 2.6 ± 0.8 | 1.7 × 10^-5^ |
| *glyA*^†,‡^ | Serine hydroxymethyl transferase | 1.1 ± 0.4 | 0.082 |
| Others |  |  |  |
|  |  |  |  |
| *fadBA*^§^ | Fatty acid β-oxidation | 3.8 ± 0.6 | 3.3 × 10^-5^ |
| *glnA*^§^ | Glutamine synthetase; nitrogen assimilation | 3.3 ± 1.0 | 0.0038 |
| *csrA*^†^ | Carbon storage regulator; RNA binding protein | 2.6 ± 0.6 | 2.0 × 10^-6^ |
| *yncE*^†,‡^ | Putative periplasmic protein; unknown function | 1.9 ± 0.3 | 0.010 |
| *yqhE*^†,‡^ | Putative 2,5-diketo-D-gluconate reductase A | 1.8 ± 0.4 | 0.012 |
| *adk*^†^ | Adenylate kinase | 1.6 ± 0.2 | 0.0038 |
| *ppa*^†,‡^ | Inorganic pyrophosphatase | 1.6 ± 0.3 | 0.13 |
|  |  |  |  |
|  | | |  |

^a^ All reporters represent promoter::*luxCDABE* fusions in pCS26 [1] generated by ^†^ PCR amplification of wild-type *S.* Typhimurium genomic DNA or ^§^ selection from a random promoter library[1]. ^‡^ Corresponding proteins including GlpX, YrbC, OppA, GltI, MglB, UgpB, DppA, RbsB, and GcvT were identified by MALDI-TOF proteomic analysis of wild-type rdar morphotype colonies (AP White, W. Kim, MG Surette, unpublished).

^b^ Descriptions were taken from the EcoCYC^TM^ database ([www.ecocyc.org](http://www.ecocyc.org/)) or from references listed in the main text.

^c^ Fold-increase (wild-type*/csgD* mutant) was calculated from the maximum CPS values of wild-type and *csgD* mutant reporter strains paired within each experiment. The average fold-increase and standard deviation from at least four biological replicates are shown.

^d^ *P* values were calculated using paired Student’s *t*-Tests with two-tailed distribution.
