# Supplementary material for: A Global Metabolic Shift Is Linked to Salmonella Multicellular Development
Source: PLoS One. 2010 Jul 27;5(7):e11814. doi: 10.1371/journal.pone.0011814 (PMC2910731; doi:10.1371/journal.pone.0011814)
Supplement: Table S2 — PCR primers used for reporter construction or mutagenesis. (0.21 MB DOC) [file pone.0011814.s002.doc]

**Table S2. PCR primers used for reporter construction or mutagenesis.**

| Primer name^a^ | Sequence |
| --- | --- |
| aceBA1 | GATCCTCGAGTCACATGAATCCAACCCTGG |
| aceBA2 | GATCGGATCCGATTCATCATGCAGCTCC |
| adk1 | GCCTCTCGAGAAGATCCGAACCAGTTTAT |
| adk2 | GCCTGGATCCTCTCCATGATGAACTGAG |
| argT1 | GCCCTCGAGCGCATAGCGTTGTCTGTT |
| argT2 | GCCGGATCCGCGAGAACGGTCTTCTTCAT |
| cadBA1 | GCCTCTCGAGTAAATTTAACGCTGAACC |
| cadBA2 | GCCTGGATCCTTTTTGACAGAACTCATG |
| csrA1 | GCCCTCGAGTGTACAAGGCTGGGTCAG |
| csrA2 | GCCGGATCCTCATCGCCAATCATGAGG |
| dppA1 | GCCCTCGAGATTTTGTGACATATTCGATGG |
| dppA2 | GCCGGATCCAGGAAATACTCATTCTTCTGC |
| fbaB1 | GATCCTCGAGGTTTAAAGGTCGTTTTCA |
| fbaB2 | GATCGGATCCACTGCGCAATATCAGTCA |
| fbp1 | GCCTCTCGAGTGAAAGTGCCACAAATTC |
| fbp2 | GCCTGGATCCATTTTATTGCCGACAGCAAAGCAGTGAGC |
| fliY1 | GCCCTCGAGGATATATATTAAAAAAGC |
| fliY2 | GCCGGATCCAGTGCTAATTTCATAGTAACC |
| gcvT1 | GCCCTCGAGCAATGGCTTCGTTAAGCG |
| gcvT2 | GCCGGATCCTACAAAGGCGTCTGTTGGG |
| glpFKX1 | GCCTCTCGAGCACTTCTAAAGACATGAT |
| glpFKX2 | GCCTGGATCCTGATGTTTGACTCATAATCG |
| gltI1 | GCCCTCGAGCGACGGAAATAATCTCTC |
| gltI2 | GCCGGATCCTCCAACTCCTTTATCATCTGC |
| glyA1 | GCCCTCGAGTTGTGCGTCAAGCATACG |
| glyA2 | GCCGGATCCTCATTTCACGCTTTAACATCC |
| gpmA1 | AGTCCTCGAGAATTATCTGGCGGGTACACC |
| gpmA2 | AGTCGGATCCGACTTTCACCGTGACGTAC |
| gshA1 | AGTCCTCGAGCTGTTAGCGGGATGGATGCG |
| gshA2 | AGTCGGATCCCTCTAACCCGCGCTGTATCC |
| kdpFABC1 | GCCTCTCGAGTGTTCCATACGTTTCCCC |
| kdpFABC2 | GCCTGGATCCAAAAATCCTTGCGCGGCCATCAGAACG |
| lsrA1 | GCCCTCGAGTATCGCTCATTGTCATAACC |
| lsrA2 | GCCGGATCCGTATTGTGACTGATTTGC |
| mglB1 | GCCCTCGAGGATCACTAACTGATTACG |
| mglB2 | GCCGGATCCTCAGTACCTTCTTATTCATGG |
| oppA1 | GCCCTCGAGAACTAAACTCCCACCTGC |
| oppA2 | GCCGGATCCGTCACTGCTTTTGCTATCACC |
| osmY1 | GCCCTCGAGAAAACGCGCGAACACTGATCC |
| osmY2 | GCCGGATCCGTCTTGTCATAGTCATCG |
| otsBA1 | GATCCTCGAGGAACAGGCTGAACATATC |
| otsBA2 | GATCGGATCCATACGGTTAACGGTTCTGC |
| pckA-A | tactattttcggtaatatatattggctaaggagcagtgat  gtgtaggctggagctgcttc |
| pckA-B | gccggatgacgccgttcaaagccgccatccggcctgttcc  catatgaatatcctccttag |
| pckA-C | atctatgagccttgtcgcggttaac |
| pckA-D | aggcataagaaagggaggtattacc |
| pgmI1 | AGTCCTCGAGAACAGTAACGCAATCCAGCC |
| pgmI2 | AGTCGGATCCAGAATCACCAGTACCATAGG |
| ppa1 | GCCCTCGAGACCCGGACGCGTAATTTT |
| ppa2 | GCCGGATCCAGTAAGCTCATGTCTGTTTCC |
| ppsA-A | gtttcttaaactcgttcatttatcacaaaaggattgttcg  gtgtaggctggagctgcttc |
| ppsA-B | attgccggatggcgacgttaacgccgccatccgtattaca  catatgaatatcctccttag |
| ppsA-C | gtttaagtatgccaggataaatacgc |
| ppsA-D | gatattattgagcggctgcgccag |
| proVWX1 | GCCTCTCGAGAGACTGGAATTTTTAACCTTACGG |
| proVWX2 | GCCTGGATCCCTAATTTAATTGCCATGC |
| rbsB1 | GCCCTCGAGTTTAACCTCCCATCAACATGC |
| rbsB2 | GCCGGATCCTGCTCCACTAGCGAAACG |
| soxS1 | GCCCTCGAGTTTTAAACGGGGAGATTT |
| soxS2 | GCCGGATCCGTCTGAATTATCTGCTGATGC |
| speA1 | GCCTCTCGAGCGATGTTATACCCATCTC |
| speA2 | GCCTGGATCCATAGACATGTCGTCAGAC |
| speB1 | GCCTCTCGAGAAGGCCTCTTAACTGACC |
| speB2 | GCCTGGATCCTGACCTAAGGTGCTCATGG |
| speC1 | GCCTCTCGAGAATTACCACAATGACTCC |
| speC2 | GCCTGGATCCATATTCATTGATTTCATAAGCG |
| STM0402-1 | GCCCTCGAGGTGACGGCCTGATAGCGA |
| STM0402-2 | GCCGGATCCCCTGACGAGTAACCAGTACC |
| ugpB1 | GCCCTCGAGTGCCTGAGACGTTAATCC |
| ugpB2 | GCCGGATCCTATGTCGTAACGATATCATCG |
| wraB1 | GCCCTCGAGTTGCCATTACTGACCTCC |
| wraB2 | GCCGGATCCAGAGCACCAGAATCTTTGC |
| yghA1 | GCCCTCGAGCAAGAAAAATAACCTTCATGG |
| yghA2 | GCCGGATCCGTATAGATGAGACATTGA |
| yncE1 | GCCCTCGAGGGAAATAATTTTCATGAA |
| yncE2 | GCCGGATCCGAAAAAAGATGACGTAAGTGC |
| yqhE1 | GCCCTCGAGGGATTTATGAAGCGGCGC |
| yqhE2 | GCCGGATCCTTAGCCATGATGTGTTCC |
| ^a^ All 1 primers contain a *Xho*I restriction site; all 2 primers contain a *Bam*HI restriction site. A and B primers were used for construction of gene knockouts in wild-type *S.* Typhimurium; C and D primers were used to confirm gene deletions in the chromosome. | |
