# Supplementary material for: A Global Metabolic Shift Is Linked to Salmonella Multicellular Development
Source: PLoS One. 2010 Jul 27;5(7):e11814. doi: 10.1371/journal.pone.0011814 (PMC2910731; doi:10.1371/journal.pone.0011814)
Supplement: Text S1 — (0.12 MB DOC) [file pone.0011814.s003.doc]

**Supporting TEXT S1**

*Transcriptional differences between wild-type* S. *Typhimurium and* csgD *mutant cultures.*

CsgA, the main structural subunit of curli, is comprised of 16% glycine[2]. The production of abundant curli at the time of aggregation could create a strong demand for intracellular glycine. The primary source of intracellular glycine in *Salmonella* is from serine in a reaction catalyzed by serine hydroxymethyl transferase, GlyA. Transcription of *glyA* was previously shown to be positively regulated by CsgD [3]. In our experiments, however, *glyA* expression was not up-regulated in wild-type cultures relative to *csgD* mutant cultures (Table S1). This could indicate that glycine is prevalent in the tryptone growth media, causing an overall decrease in *glyA* transcription, as shown for *E. coli* grown in media supplemented with amino acids [3,4]. Consistent with this hypothesis, transcription of the *gcvTPH* operon, encoding enzymes of the glycine cleavage complex, was up-regulated in wild-type cells coinciding with aggregation (Table S1). It is thought that the glycine cleavage complex may be required to generate C1 units for biosynthesis of cellular components[5], which could contribute to the resources needed for aggregation.

*CsrA* encodes an RNA binding protein (carbon storage regulator) with an important regulatory role in central carbon flux, carbon storage, motility and biofilm formation[6]. Transcription of *csrA* was significantly induced in wild-type cultures at the time of aggregation (Table S1). This result was unexpected since up-regulation of CsrA was previously shown to inhibit biofilm formation[7]. However, CsrA activity is influenced by two non-coding, antagonist RNAs[6] and without knowing their expression it is difficult to draw conclusions. One possibility is that CsrA is involved in regulating central carbon flux during aggregation of wild-type *S.* Typhimurium.

The increased levels of octanoic acid detected by ^1^H NMR in wild-type colonies was difficult to explain. Expression of *fadBA*, encoding enzymes required for fatty acid β-oxidation[8], was significantly induced in wild-type cells (Table S1) and timed with aggregation (data not shown). We reasoned that increased fatty acid oxidation may be necessary to help funnel carbon into gluconeogenesis. *fadBA* expression was previously shown to be up-regulated in *E. coli* during carbon starvation[9], suggesting that a similar phenomenon may be occurring here.

One of the key signals that stimulates *csgD* transcription and subsequent rdar morphotype aggregation is nitrogen limitation[10]. There are only three central metabolites for nitrogen assimilation into *S.* Typhimurium, one precursor, 2-oxoglutarate, and two intermediates, glutamate and glutamine, from which nitrogen is incorporated into other compounds [11]. The first signal of nitrogen limitation is a drop in the intracellular level of glutamine coupled with an increase in expression of glutamine synthase (GlnA) [11]. GlnA catalyzes the formation of glutamine from glutamate and ammonia in a reaction requiring ATP. We analyzed the expression of *glnA* because this gene was shown to be induced in related gene expression studies (JS Happe, RJ Martinuzzi, V Kostenko, MG Surette, submitted). *GlnA* was up-regulated in wild-type cultures (Table S1) relative to *csgD* mutant cultures and the peak expression was timed with aggregation (data not shown). Increased GlnA expression could potentially explain the elevated levels of pyroglutamate detected in wild-type colonies. The spontaneous conversion of glutamine to pyroglutamate could easily have occurred during the GC-MS derivatization process[12]. These results suggest that aggregating wild-type cells may be nitrogen-limited compared to planktonic *csgD* mutant cells.

Carnitine, a quaternary ammonium compound formed from lysine and methionine, occurs ubiquitously in nature due to its crucial role in β-oxidation of fatty acids in mitochondria[13]. Some bacteria are able to utilize carnitine as a carbon or nitrogen source, however *E. coli* cells accumulate it as an osmoprotectant by importation via the ProP and ProU systems[13]. Galactinol, an oligosaccharide formed from galactose and myo-inositol, was recently shown to act as both an osmoprotectant and antioxidant[14] in plants. Little is known about either of these compounds in the context of *Salmonella* biology. We hypothesize that carnitine and galactinol are present in T agar and were accumulated to function as osmoprotectants in wild-type *S.* Typhimurium cells.

Additional genes up-regulated in the wild-type strain included *ppa* [15] and *yncE* and *yqhE,* two genes that encode proteins with unknown functions; YncE and YqhE were identified by proteomics in rdar morphotype colonies (data not shown).

**supporting information references**

1. Bjarnason J, Southward CM, Surette MG (2003) Genomic profiling of iron-responsive genes in *Salmonella enterica* serovar Typhimurium by high-throughput screening of a random promoter library. J Bacteriol 185: 4973-4982.

2. Collinson SK, Parker JM, Hodges RS, Kay WW (1999) Structural predictions of AgfA, the insoluble fimbrial subunit of *Salmonella* thin aggregative fimbriae. J Mol Biol 290: 741-756.

3. Chirwa NT, Herrington MB (2003) CsgD, a regulator of curli and cellulose synthesis, also regulates serine hydroxymethyltransferase synthesis in *Escherichia coli* K-12. Microbiology 149: 525-535.

4. Brombacher E, Baratto A, Dorel C, Landini P (2006) Gene expression regulation by the Curli activator CsgD protein: modulation of cellulose biosynthesis and control of negative determinants for microbial adhesion. J Bacteriol 188: 2027-2037.

5. Meedel TH, Pizer LI (1974) Regulation of one-carbon biosynthesis and utilization in *Escherichia coli*. J Bacteriol 118: 905-910.

6. Babitzke P, Romeo T (2007) CsrB sRNA family: sequestration of RNA-binding regulatory proteins. Curr Opin Microbiol 10: 156-163.

7. Jackson DW, Suzuki K, Oakford L, Simecka JW, Hart ME, et al. (2002) Biofilm formation and dispersal under the influence of the global regulator CsrA of *Escherichia coli*. J Bacteriol 184: 290-301.

8. Iram SH, Cronan JE (2006) The beta-oxidation systems of *Escherichia coli* and *Salmonella enterica* are not functionally equivalent. J Bacteriol 188: 599-608.

9. Liu M, Durfee T, Cabrera JE, Zhao K, Jin DJ, et al. (2005) Global transcriptional programs reveal a carbon source foraging strategy by *Escherichia coli*. J Biol Chem 280: 15921-15927.

10. Gerstel U, Romling U (2001) Oxygen tension and nutrient starvation are major signals that regulate *agfD* promoter activity and expression of the multicellular morphotype in *Salmonella typhimurium*. Environ Microbiol 3: 638-648.

11. Ikeda TP, Shauger AE, Kustu S (1996) *Salmonella typhimurium* apparently perceives external nitrogen limitation as internal glutamine limitation. J Mol Biol 259: 589-607.

12. Garcia DE, Baidoo EE, Benke PI, Pingitore F, Tang YJ, et al. (2008) Separation and mass spectrometry in microbial metabolomics. Curr Opin Microbiol 11: 233-239.

13. Verheul A, Wouters JA, Rombouts FM, Abee T (1998) A possible role of ProP, ProU and CaiT in osmoprotection of *Escherichia coli* by carnitine. J Appl Microbiol 85: 1036-1046.

14. Nishizawa A, Yabuta Y, Shigeoka S (2008) Galactinol and raffinose constitute a novel function to protect plants from oxidative damage. Plant Physiol 147: 1251-1263.

15. Lahti R, Pohjanoksa K, Pitkaranta T, Heikinheimo P, Salminen T, et al. (1990) A site-directed mutagenesis study on *Escherichia coli* inorganic pyrophosphatase. Glutamic acid-98 and lysine-104 are important for structural integrity, whereas aspartic acids-97 and -102 are essential for catalytic activity. Biochemistry 29: 5761-5766.
